# Degradation and modification of cochlear gap junction proteins in the early development of age-related hearing loss

**DOI:** 10.1038/s12276-020-0377-1

**Published:** 2020-01-27

**Authors:** Shori Tajima, Keiko Danzaki, Katsuhisa Ikeda, Kazusaku Kamiya

**Affiliations:** Department of Otorhinolaryngology, Juntendo University Faculty of Medicine, Tokyo, 113–8421 Japan

**Keywords:** Experimental models of disease, Protein quality control

## Abstract

Age-related hearing loss (ARHL) is the progressive, bilateral loss of high-frequency hearing in elderly people. Mutations in *GJB2*, encoding the cochlear gap junction protein connexin26 (Cx26), are the most frequent cause of hereditary deafness; however, a common molecular pathology between ARHL and *GJB2*-related hearing loss has not been reported. Here, we investigated the quantitative change in expression and molecular pathology of Cx26 in ARHL. We used C57BL/6J mice as a model of ARHL. Hearing levels that were evaluated by auditory brainstem response thresholds increased gradually between 4 and 32 weeks of age and increased sharply at 36 weeks. Gap junctions in the cochleae of 4-week-old mice had linear plaques along cell–cell junction sites. In contrast, the cochleae from 32-week-old mice had significantly shorter gap junctions. Severe hair cell loss was not observed during this period. Based on western blotting, Cx26 and connexin30 (Cx30) levels were significantly decreased at 32 weeks compared with 4 weeks.

Moreover, Cx26 was more significantly enriched in the hydrophilic fraction at 4 weeks but was more significantly enriched in the hydrophobic fraction at 32 weeks, indicating an age-related conversion of this biochemical property. Thus, the hydrophobic conversion of Cx26 and disruption of gap junction proteins and plaques may be involved in the pathogenesis of ARHL and may occur before severe hair cell degeneration.

## Hearing loss: Disruption at the junction

A decrease in the levels of connexin proteins at the junctions connecting cells in the inner-ear precedes age-related hearing loss (ARHL) in mice. Loss of hearing in the elderly is a growing problem in ageing populations. Although mutations in genes encoding connexins have been associated with hereditary hearing loss, their role in ARHL is poorly understood. Kazusaku Kamiya and colleagues at Juntendo University, Tokyo, found that the levels of connexin 26 and connexin 30 were significantly reduced in the cochlea in the inner ear of 32-week old mice compared to 4-week old mice. Connexin 26 also became less soluble with age. The authors suggest that these changes could lead to the degeneration and loss of function of hair cells in the cochlea, and that targeting connexin 26 could lead to new therapies for ARHL.

## Introduction

Age-related hearing loss (ARHL), also known as presbycusis, is a growing problem because of the increase in the elderly population. Hearing loss of all types affects not only communication but also quality of life. Recent evidence suggests that hearing loss may be an early sign of and contributor to dementia^1^. The use of hearing aids is recommended as a treatment for ARHL, but there are currently no treatments that can restore hearing.

The mechanisms of aging in the auditory system have been the subject of investigation for decades. It has been proposed that ARHL can be explained largely by the degeneration of the lateral wall of the cochlea, which includes the stria vascularis, minor losses of the outer and inner hair cells (OHCs and IHCs, respectively), and some loss of spiral ganglion cells^2^. Strial atrophy is well recognized in aged human temporal bones^3^. The loss of OHCs and IHCs raises the issue of noise damage as a confounding factor rather than a main contributor to auditory aging. Currently, the early-stage pathologies of ARHL are not fully understood.

Supporting cells are extensively coupled by large gap junctions, which are one of the most important pathways for communication between adjacent cells to maintain hearing function^4,5^. K^+^ is removed through these gap junctions from the base of hair cells to the endolymph, and the returning K^+^ maintains a high endocochlear potential (EP), which allows hair cells to depolarize and send signals to the cochlear nerve^6^. However, a recent study suggested that K^+^ recycling was not essential in the connexin26 (Cx26) deficiency deafness mechanism, although K^+^ recycling via gap junctions plays a critical role in hearing^7^. To control hearing sensitivity, OHC electromotility, which serves as an active cochlear amplifier, also plays an important role^8,9^. We demonstrated that a dominant-negative CX26-R75W transgenic mouse model has an incomplete development of the cochlea, and supporting cells showed no detectable distortion-product otoacoustic emissions despite normal development and electromotility of OHCs^10^. Moreover, Zhu et al. demonstrated that active cochlear amplification was dependent on supporting cell gap junctions and that Cx26 deficiency might impair active cochlear amplification leading to late-onset hearing loss^11,12^. Thus, cochlear supporting cells and gap junctions are one of the most important structures in active cochlear amplification.

Cx26 and connexin30 (Cx30), which are encoded by gap junction proteins beta-2 and beta-6 (*GJB2* and *GJB6*, respectively), are the major protein subunits in cochlear gap junctions, and mutations of *GJB2* are the most typical cause of hereditary sensorineural deafness^13^. We previously reported that a mutation in Cx26 induces macromolecular degradation of large gap junction complexes and that the assembly of these complexes requires Cx26^14^. In addition, *Brn4*-deficient mice, used as a model of deafness type 3 (DFN3) nonsyndromic deafness, have disrupted and scattered gap junction plaques (GJPs) compared to those in control mice, as well as significantly reduced levels of Cx26 and Cx30^15^. Taken together, these studies suggest that the degradation of GJPs may result in a reduction in cochlear amplification and severe hearing loss.

The C57BL/6J mouse has been suggested as an optimal animal model of ARHL^16–18^. C57BL/6J mice maintain normal hearing for 13 weeks. After that, high-frequency hearing loss is observed, becoming profound at 30 weeks at the latest, with the cutoff frequency decreasing gradually with advancing age^19^. Significant hearing loss across all frequencies has been reported by 65 weeks, with no measurable auditory response at 78 weeks^20^.

There is a significantly reduced density of Cx26 in the spiral ligament in aged C57BL/6J mice, suggesting the possibility of a substantial disruption in gap junction connections among fibrocytes^21^. It was later proposed that two mild mutations in *GJB2* might be associated with an increased risk of early presbycusis in humans compared with control individuals^22^. Moreover, Fetoni et al. reported that the hearing function worsened more rapidly in *GJB2*^+/−^ mice compared to control mice, indicating that they were affected by accelerated ARHL^23^.

Furthermore, the aging process is thought to be associated with damage to mitochondria by reactive oxygen species^24^. Increases in reactive oxygen species production are associated with changes in the expression or function of gap junctions and affect connexin expression^25^. Deletions in antioxidant enzymes can also lead to ARHL^26^. Thus, Cx26 may be affected by oxidative stress in the cochlea, contributing to ARHL, and dysfunction of cochlear gap junctions may be related to the development of presbycusis.

Here, we hypothesized that disruption in cochlear GJPs may be involved in ARHL and investigated the quantitative and biochemical changes in Cx26 and Cx30 as a means of understanding the molecular pathology of the early stage of ARHL in the C57BL/6J mouse model.

## Materials and methods

### Animals and ethics statement

The care, maintenance, and treatment of animals in this study followed protocols approved by the Institutional Animal Care and Use Committee at Juntendo University (Permit Number: 310138). All research was performed in accordance with relevant guidelines and regulations. Cx26 knockout mice were obtained from a breeding colony of a previously reported mouse line^14^. C57BL/6J mice were chosen as an animal model for ARHL because they exhibit progressive hearing loss in middle age. The C57BL/6J mice used in this study were purchased from Charles River Laboratories (Yokohama, Japan). Animals were used for experiments at 4 weeks (*n* = 17 mice) and 32 weeks (*n* = 17 mice) of age.

### Auditory brainstem response

All recordings were performed in a soundproof chamber with an electrostatic shield to avoid electrical noise. For the auditory brainstem response (ABR) measurements, stainless-steel needle electrodes were placed at the vertex and ventrolateral to the left and right ears. ABRs were measured using the waveform storing and stimulus control in the Scope software of the PowerLab system (PowerLab 4/25; AD Instruments), and electroencephalogram recordings were made with an extracellular amplifier AC PreAmplifier (model P-55; Astro-Med). Acoustic stimuli were delivered to mice through a coupler-type speaker (model ES1spc; Bio Research Center). The threshold was determined at frequencies of 8, 20, and 40 kHz from a set of responses at varying intensities with 5-dB intervals.

### Light microscopy

Animals were anesthetized and then intracardially perfused with 0.01 M phosphate-buffered saline (PBS; pH 7.2), followed by 4% paraformaldehyde (pH 7.4) in PBS. The mice were decapitated, and their cochleae were dissected under a microscope. Dissected cochleae were placed in 4% PFA for 1 hour at 4 °C. Cochleae were then placed in 0.12 M ethylenediaminetetraacetic acid (EDTA; pH 7.0) in PBS for 1 week for decalcification. The specimens were then dehydrated, embedded in paraffin and sectioned (6 μm). Serial sections were stained with hematoxylin and eosin staining.

### Immunohistochemistry

Mice were anesthetized and killed before the inner-ear tissues were removed. Cochleae were dissected and fixed in 4% paraformaldehyde in phosphate-buffered saline (PBS). Immunofluorescence staining with mouse anti-Cx26 (1:300; Invitrogen #33–5800), rabbit anti-Cx30 (1:200; Invitrogen #71–2200), Alexa Fluor 488-conjugated phalloidin (1:500; Invitrogen #A12379) and Alexa Fluor 555-conjugated cholera toxin subunit B (CTxB, 1:1000; Invitrogen #C34776) was performed on whole-mount preparations of finely dissected organs of Corti, which included the ISCs. To validate the antibodies used, we performed validation experiments by expressing recombinant connexins in connexin-free HeLa cells and HeLa cells with a stable expression of Cx26 and Cx30 (data not shown). Tissues were incubated in antibody solutions for 1 h at room temperature after blocking with 2% bovine serum albumin in 0.01 M PBS. Confocal fluorescence images were obtained using an LSM780 confocal microscope (Carl Zeiss), which has an inverted configuration with an objective lens with a numerical aperture of 0.8 and 1.2 in low- (×20) and high-power (×63) fields, respectively. Cx26, Cx30, and hair cells were immunolabeled using Alexa Fluor 488-conjugated mouse IgG, rabbit IgG and phalloidin (Life Technologies; green fluorescence #ab150113 and #ab150077), respectively and were observed by using the 488-nm line of the confocal microscope. Lipid rafts were immunolabeled with CTxB (red fluorescence) and observed by using the 555-nm line of the confocal microscope. We observed cochlear GJPs in ISCs and hair cells in the same region of the apical middle turn, with a 35–45% estimated distance from the apex. The *z*-stacks of images were collected at 0.5-μm intervals, and the single image stacks were constructed with an LSM Image Browser (Zeiss). Three-dimensional images were constructed with *z*-stacked confocal images by IMARIS (Bitplane). Quantitative analysis of the GJP length (mean ± SE) was performed using an LSM Image Browser (Carl Zeiss). The lengths of GJPs from 12 cochleae from six mice for each group were measured, and the distribution of the GJP size was obtained and represented by using a box-and-whisker plot. The mean GJP length was compared using Student’s *t*-test (Microsoft Excel).

### Western blotting

Mouse cochlear proteins were extracted with RIPA buffer (Nacalai Tesque) from at least 10 mice (2 cochleae each), including the organ of Corti, lateral wall, and stria vascularis. Proteins were resolved by sodium dodecyl sulfate-polyacrylamide gel electrophoresis using mini-PROTEAN TGX gradient gels (4–20% polyacrylamide; Bio-Rad Laboratories) and transferred to polyvinylidene difluoride membranes (Trans-Blot Turbo; Bio-Rad Laboratories). After blocking with 4% Block Ace (DS Pharma Biomedical Co., Ltd.) in Tris-buffered saline with Tween 20 (Tokyo Chemical Industry), the membranes were processed through sequential incubations with mouse anti-Cx26 (1:1500), rabbit anti-Cx30 (1:1000), and mouse monoclonal anti-β-actin (1:2000; Sigma). Horseradish peroxidase-conjugated anti-mouse IgG (1:15,000; GE Healthcare) and anti-rabbit IgG (1:10,000; GE Healthcare) were used as secondary antibodies. Amersham ECL Prime Western Blotting Detection Reagent (GE Healthcare) was used for visualization. Signals were observed, and densitometric analyses were performed on an Amersham Imager 600 (GE Healthcare). Each experiment was carried out at least three times. Data were normalized to the corresponding β-actin levels and expressed relative to the levels in each control, and comparisons were conducted using Student’s *t*-test (Excel).

### Real-time RT-PCR

Total RNA was isolated from six cochleae, which were dissected out carefully under a microscope using an RNeasy Plus Mini Kit on a QIAcube instrument (Qiagen). Then, the RNA was reverse transcribed to cDNA with a PrimeScript II 1st strand cDNA Synthesis Kit (Takara), and the cDNA was submitted for quantitative real-time PCR with TB Green Premix Ex Taq II (Takara) to determine the gene expression using the 2^−ΔΔCT^ method. The primer sequences for mouse GAPDH, Cx26, and Cx30 used in this study were: GAPDH forward: 5′-TGTGTCCGTCGTGGATCTGA-3′; GAPDH reverse: 5′-TTGCTGTTGAAGTCGCAGGAG-3′; Cx26 forward: 5′-ACTGGAAGCGTCTCGTGCTG-3′; Cx26 reverse: 5′-GGCAGTTGTCGGCATATCCTATC-3′; Cx30 forward: 5′-CCCAATCTCGTGGACTGCTTC-3′; and Cx30 reverse: 5′-GTAACACAACTCGGCCACATTGA-3′.

### Separation of the hydrophilic and hydrophobic fractions of cochlear proteins

The hydrophilic and hydrophobic fractions of the total cochlear proteins were separated using the Cellytic MEM Protein Extraction Kit (Sigma-Aldrich), which is based on liquid–liquid phase separation (LLPS). Proteins were detected by western blotting with mouse anti-Cx26 (1:200) and rabbit anti-Cx30 (1:300) with horseradish peroxidase-conjugated anti-mouse IgG (1:10,000) or anti-rabbit IgG (1:10,000) as secondary antibodies. Western blotting was performed for each mouse (2 cochleae) without grouping. Densitometric analysis of band intensities was performed on the Amersham Imager 600. Each experiment was carried out at least three times. Data in (Fig. 6b, c) were normalized by using whole-cell lysates.

## Results

### Progression of hearing loss in aging C57BL/6J mice

**Fig. 1:**
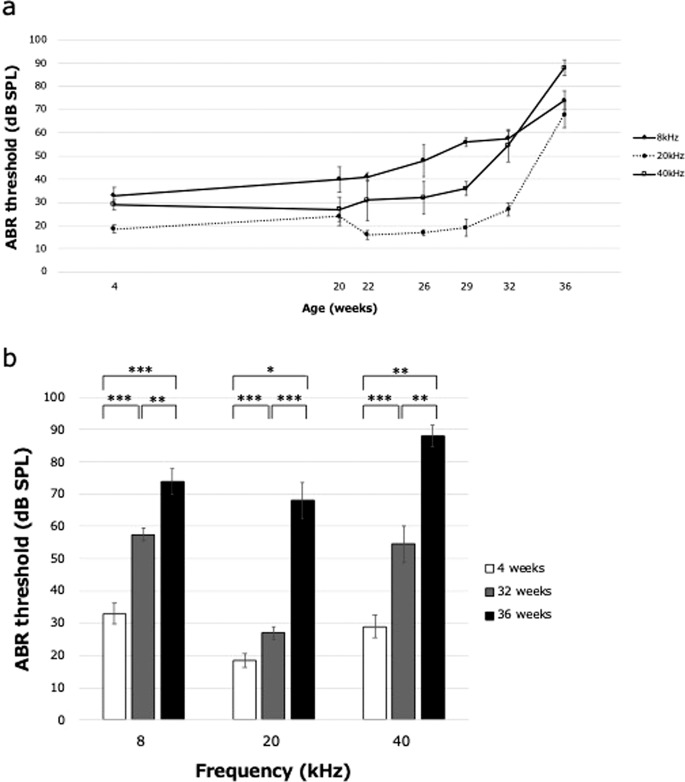
High-frequency hearing loss in C57BL/6J mice measured as changes in the ABR threshold. **a** At all frequencies tested, there was no obvious hearing loss prior to 26 weeks, but there was some hearing loss at 32 weeks and greater hearing loss at 36 weeks. **b** ABR recordings show progressive elevated threshold with age. Values represent the mean ± standard error (SE). *n* = 10 mice at both 4 and 32 weeks and *n* = 5 mice at 36 weeks. *P < 0.05, **P < 0.01, ***P < 0.001, one-way ANOVA with Bonferroni’s method. SPL sound pressure level.

To assess changes in the hearing of C57BL/6J mice with age, ABR thresholds were recorded at 4 weeks of age and then monitored regularly from 20 to 36 weeks of age (Fig. 1a). At all frequencies tested, the ABR threshold increased slightly at 32 weeks and increased sharply at 36 weeks. At each frequency, the ABR threshold was significantly elevated in 32-week-old mice compared with 4-week-old mice and was even more significantly increased at 36 weeks (Fig. 1b, Supplementary Fig. 1). These data suggest that the pathological progression of hearing loss at all frequencies began prior to 32 weeks of age and accelerated between 32 and 36 weeks.

### Changes in GJPs during early ARHL

**Fig. 2:**
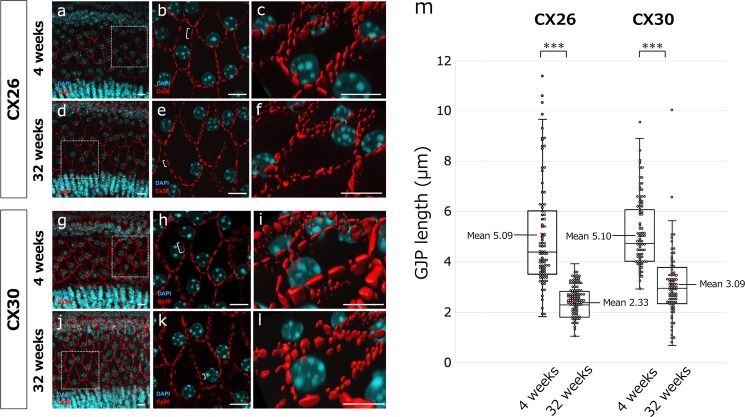
Disruption of GJPs in 4- and 32-week-old mice. GJPs were immunolabeled for Cx26 and Cx30 (red), and nuclei were counterstained with DAPI (blue). **a–l** *z*-Stack images of Cx26 (**a–f)** and Cx30 (**g–l**) of cochlear ISCs from 4- (**a–c** and **g–i**) and 32-week-old (**d–f** and **j–l**) mice were obtained by confocal microscopy. (**c**, **f**, **i**, **l**) Three-dimensional images were reconstructed from *z*-stacks to show a detailed structure. **m** The length of the largest GJP along a single cell border was measured in the *z*-stack confocal images, and the length distribution was analyzed by using a box-and-whisker plot. Red x indicates the mean of the GJP lengths. (*n* = 83 GJPs in six 4-week-old mice, *n* = 100 GJPs in six 32-week-old mice for Cx26, *n* = 91 GJPs in six 4-week-old mice, and *n* = 79 GJPs in six 32-week-old mice for Cx30). ***P < 0.001 (Student’s *t*-test). Scale bars indicate 10 μm.

**Fig. 3:**
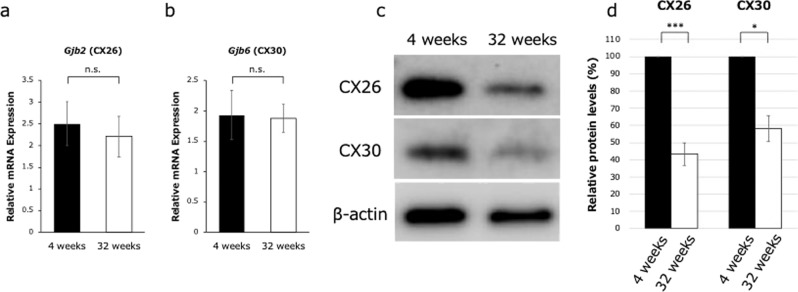
GJP protein levels were reduced in 32-week-old mice without a reduction in mRNA expression levels. **a**, **b** Cx26 and Cx30 mRNA expression levels were not significantly different between 4- and 32-week-old mice. Values are expressed relative to the amount at 4 weeks of age and represent the mean ± SE (*n* = 6 mice at 4 weeks, *n* = 5 mice at 32 weeks). **c** Total amount of cochlear Cx26 and Cx30 protein levels were assessed in 4- and 32-week-old mice by western blotting. **d** Blots were quantified, and connexin levels were normalized to those of β-actin (*n* = 6 mice at 4 weeks, *n* = 4 mice at 32 weeks). *P < 0.05, ***P < 0.001 (Student’s *t*-test); n.s. not significant.

To assess the mechanisms of hearing loss at an earlier stage, we performed a detailed analysis of cochlear GJPs of the inner sulcus cells (ISCs) in 4- and 32-week-old mice. We first performed an immunohistochemical analysis of Cx26 and Cx30 expression in cochlear ISCs (Fig. 2). In 4-week-old mice, gap junctions formed linear structures along cell–cell junction sites, creating pentagonal or hexagonal outlines of ISCs and border cells (Fig. 2a–c–g–l). In contrast, GJPs of ISCs in 32-week-old mice did not show this normal linear structure but instead were scattered and appeared as small spots around the cell–cell junction sites (Fig. 2d–f–j–l). To quantify this difference, we measured the length of the largest GJPs with Cx26 and Cx30 along a single-cell border. The average lengths of GJPs, using Cx26 and Cx30 immunolabeling, were significantly shorter in 32-week-old mice than in 4-week-old mice (Fig. 2m), suggesting that the GJP size was reduced with age as shown in Cx26-deficient mice (Supplementary Fig. 2). To further assess this reduction, we performed western blotting for Cx26 and Cx30 proteins in the cochleae of 4- and 32-week-old mice. The levels of both proteins were significantly decreased at 32 weeks of age (57 and 42%, respectively; Fig. 3). Moreover, to assess this decrease in the protein levels, the Cx26 and Cx30 mRNA expression levels were measured by quantitative RT-PCR (qRT-PCR). As can be seen in the results, mRNA expression levels were not significantly different between 4 and 32 weeks of age. The combined confocal and quantitative analyses by western blotting and qRT-PCR suggested that the gap junction macromolecular complex had degraded between 4 and 32 weeks of age, and this degradation may be one of the causes of the observed hearing impairment in this mouse model of ARHL; however, other biological changes, such as synaptic inactivity and neuronal degeneration, are still unclear.

### The loss of hair cells is not visibly severe during early ARHL

**Fig. 4:**
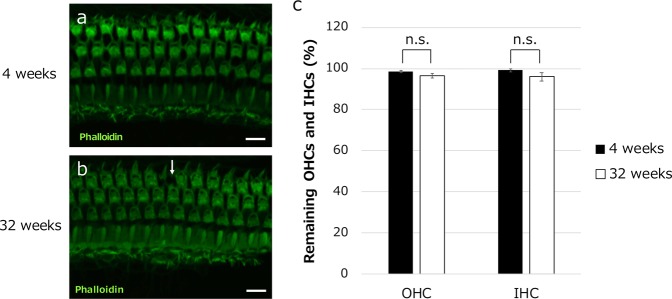
IHCs and OHCs are similar in 4- and 32-week-old mice. **a**, **b** Representative images of cochlear F-actin staining with phalloidin revealed no obvious differences in the number or morphology of OHCs and IHCs between mice at 4 and 32 weeks of age. The arrow in **b** indicates a loss of OHCs. **c** OHC and IHC survival was not significantly different between mice at 4 and 32 weeks of age. (*n* = 4 mice each.) n.s. not significant. Scale bars indicate 10 μm.

OHCs and IHCs were also compared between 4- and 32-week-old mice by staining filamentous actin with phalloidin (Fig. 4a, b). Hair cell morphology was not noticeably different between 4 and 32 weeks of age. Only small differences in the cell numbers were observed (3.6% and 4.1% for OHCs and IHCs, respectively; Fig. 4c), and these differences were not statistically significant. This indicates that hair cell loss was not significantly affected by age and may not be a likely factor in the initial stage of ARHL.

### Lipid rafts in the cochlea become diffuse with age

**Fig. 5:**
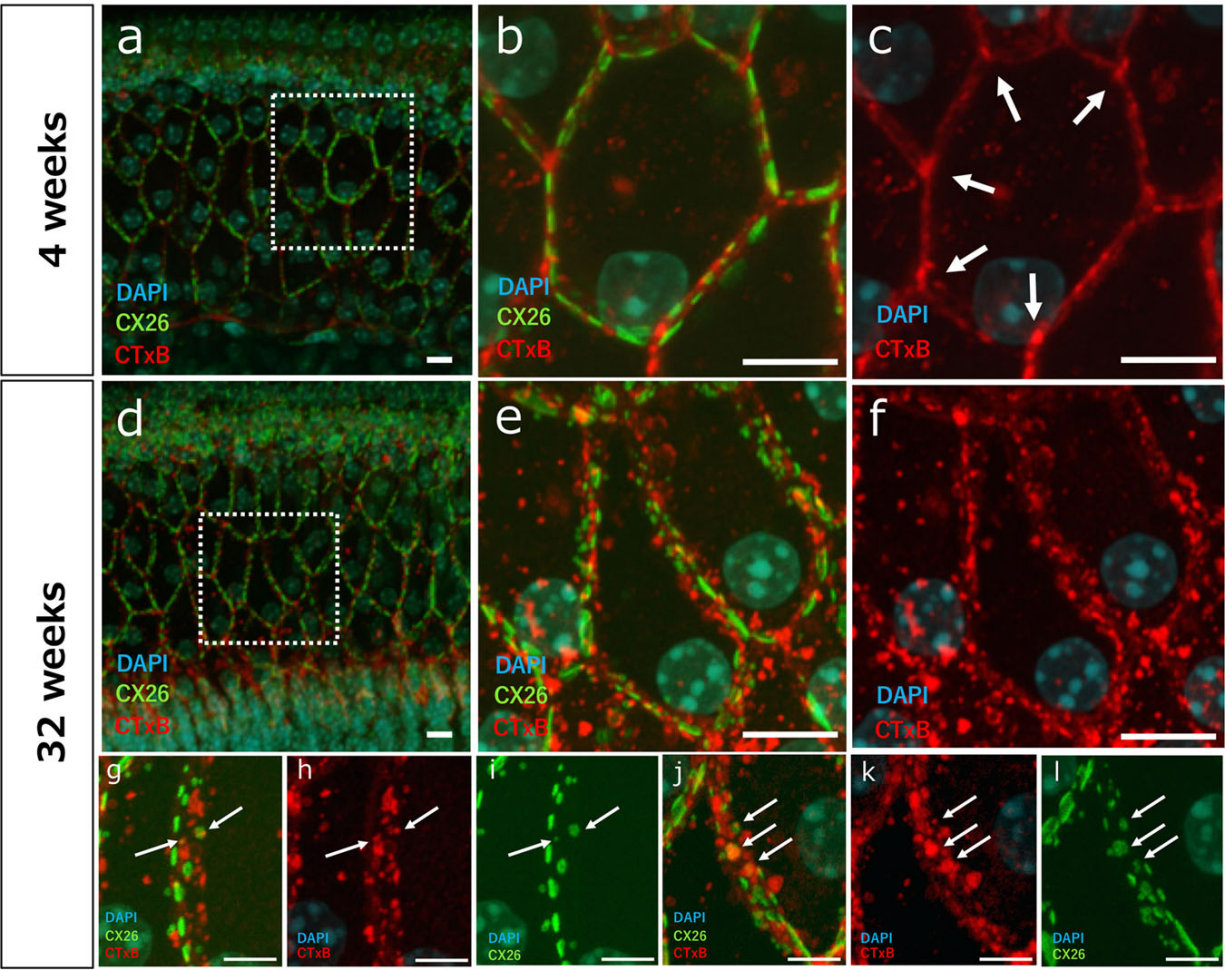
Changes in the distribution of lipid rafts around degraded GJPs of ISCs in 32-week-old mice and the process of degradation of GJPs. Lipid rafts were stained in cochleae from 4- and 32-week-old mice using CTxB (red) and were counterstained with anti-Cx26 (green) and DAPI (blue). **a**, **b** In 4-week-old mice, lipid rafts were observed between GJPs. **d**, **e** In 32-week-old mice, lipid rafts were scattered around degraded GJPs with no regularity. Focusing on the distribution of lipid rafts, in 4-week-old mice (**c**), lipid rafts localize mainly at multicellular junction sites between adjacent GJPs. Arrows in **c** indicate the accumulation of lipid rafts adjacent to normal GJPs. In 32-week-old mice (**f**), lipid rafts contributed to the irregularly around GJPs. **g**, **j** Representative images of the cell borders of cochlear supporting cells in 32-week-old mice. As Cx26 became increasingly hydrophobic with age, lipid rafts were observed around GJPs (**h** and **k**), and some GJPs were colocalized with lipid rafts (**i** and **l**) and fragmented. Arrows in (**g–l**) indicate the colocalization between lipid rafts and GJPs. Scale bars indicate 10 μm (**a–f**) and 5 μm (**g–l**).

We previously showed that diffuse labeling of lipid rafts surrounding disrupted GJPs is associated with *GJB2*-related hearing loss in Cx26-deficient mice^14^. We hypothesized that the disrupted GJPs in 32-week-old mice are associated with the unordered distribution of the lipid rafts. Here, we labeled the cochleae ISCs of 4- and 32-week-old mice with CTxB to visualize lipid rafts. In 4-week-old mice, lipid rafts were localized in an orderly fashion between the GJPs and were not colocalized with GJPs (Fig. 5a–c). In 32-week-old mice, the lipid raft signals were distributed in a diffuse and disorderly fashion around the GJPs (Fig. 5d-f). Moreover, focusing on the distribution of GJPs and lipid rafts, some GJPs were colocalized with lipid rafts in 32-week-old mice (Fig. 5g–l).

### Cx26 becomes more hydrophobic with age

**Fig. 6:**
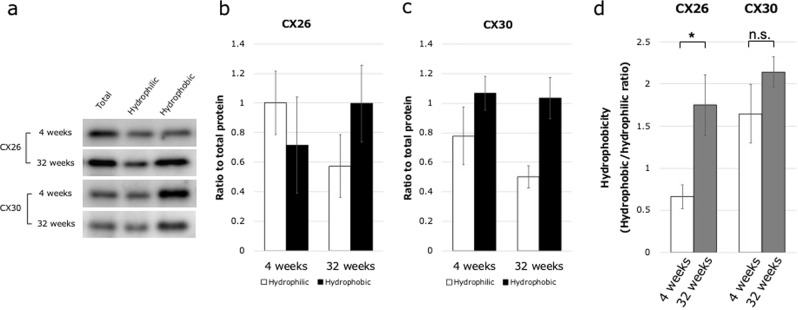
Biochemical change in Cx26 polarity from hydrophilic to hydrophobic with age. **a** Western blots of Cx26 and Cx30 in total, hydrophilic, and hydrophobic fractions of cochlear membrane proteins from 4- and 32-week-old mice. **b**, **c** Biochemical change with age in Cx26 polarization from hydrophilic to hydrophobic fractions with normalization by using whole-cell lysates. **d** Hydrophobicity (hydrophobic/hydrophilic ratio) of Cx26 and Cx30 in cochlear proteins. **b**, **d** Cx26 was mainly in the hydrophilic fraction at 4 weeks but was substantially enriched in the hydrophobic fraction at 32 weeks, causing a significant increase in the hydrophobic/hydrophilic ratio. **c**, **d** There were no such changes for Cx30. Values represent the mean ± SE (*n* = 4 mice each). *P < 0.05 (Student’s *t*-test); n.s. not significant.

To investigate the relationship between connexins and lipid rafts, we used a biochemical approach to separate the hydrophobic and hydrophilic microdomains of cochlear membrane proteins by the LLPS method and then quantified Cx26 and Cx30 in these fractions by western blotting (Fig. 6a). In 4-week-old mice, more Cx26 protein was detected in the hydrophilic fraction than in the hydrophobic fraction (0.7-fold relative to hydrophilic; Fig. 6b–d), indicating that the Cx26 in cochleae from young mice has a hydrophilic preference. In contrast, in 32-week-old mice, there was more Cx26 protein in the hydrophobic fraction (1.7-fold relative to the hydrophilic fraction; Fig. 6b–d). In other words, the ratio of Cx26 in the hydrophobic to hydrophilic fractions increased with age (Fig. 6d), suggesting that there is an age-related change in the biochemical property of Cx26 that might be associated with the disrupted GJPs that are surrounded by lipid rafts observed in older mice. In contrast, there was no such conversion in the hydrophilic/hydrophobic nature of Cx30 with age (Fig. 6a–c, d).

## Discussion

In the ABR analysis, we found that the pathological progression of ARHL was accelerated between 32 and 36 weeks of age. The ABR thresholds of C57BL/6J mice have been investigated over the past decade. In a previous study, C57BL/6J mice exhibited a moderate high-frequency hearing loss (32 kHz) by 6 months of age that progressed to almost complete high-frequency deafness by 18 months^27^. In another study, on the other hand, hearing loss of C57BL/6J mice at a high-frequency (24 and 32 kHz) progressed gradually between 14 and 36 weeks of age, followed by significant hearing loss at 60 weeks^28^, but detailed monitoring of hearing loss between 36 and 60 weeks was not conducted. Moreover, the ABR threshold at 16 kHz in C57BL/6J mice rapidly increased after 30 weeks^29^, as shown in the present study. Although the C57BL/6J mouse line is commercially available worldwide, the auditory functions and inner-ear pathology written in the articles are not always the same. This is because hearing is not included in the quality check items in laboratory animal suppliers. In the present study, we validated that the ABR threshold of C57BL/6J mice increased slightly at 32 weeks, as described in previous reports. Then, as the early gradual pathological progression of ARHL accelerated at ~32 weeks, we analyzed the molecular pathology of cochlear GJPs in 4- and 32-week-old mice.

In our immunohistochemical study of the cochlea, we analyzed the GJPs in ISCs, rather than GJPs in other supporting cells such as Deiters’ cells, pillar cells, Hensen cells, and fibrocytes, as a representative of cochlear GJPs because, unlike the other gap-junction-forming cells, ISCs are unique in that they form a single-cell layer that enabled us to construct a clear three-dimensional GJP structure from confocal *z*-stack images. The GJP sizes of ISCs were reduced, and the gap junction proteins, such as Cx26 and Cx30, were significantly decreased in 32-week-old mice compared with 4-week-old mice. These changes were similar to what we observed in Cx26 knockout (KO) mice (Supplementary Fig. 2) and *Brn4* KO mice^15^. On the other hand, in the qRT-PCR analysis, mRNA expression showed no significant differences between 4 and 32 weeks of age. This result indicated that the decrease in the protein levels of Cx26 and Cx30 was not caused by a decrease in the mRNA expression but by the degradation or instability of GJPs. At this stage, there was no significant severe hair cell loss or other visibly obvious degeneration in the sensory epithelium (Fig. 4). The morphological alteration of hair cells with aging has previously been studied. Hequembourg et al. reported that the OHC population remained almost intact in the middle and basal turns of the cochlea of C57BL/6 J mice at 7 months of age^19^. We demonstrated that hair cell loss was not severe in 32-week-old C57BL/6J mice purchased from the same laboratory animal supplier during the same period. To assess other morphological changes in hair cells, quantitative analysis of the height of the organ of Corti was performed. There was no difference in the height of the organ of Corti between 4- and 32-week-old mice (Supplementary Fig. 3). These data suggest that cochlear gap junction degradation may be involved in the early-stage pathology of ARHL, as it occurs before severe degeneration of the sensory epithelium.

The relationship between gap junction degradation and hearing loss is still unclear. EP is essential for the cochlea to maintain the high positive potential of the endolymph, but the EP of C57BL/6J mice shows no significant differences with aging^30,31^. On the other hand, active cochlear amplification is one of the important mechanisms for amplifying acoustic stimulation and for increasing the hearing sensitivity and frequency selectivity^9,32^. Zhu suggested that the determining factor in Cx26 deficiency-induced hearing loss might be associated with not only the reduction in the EP but also the reduction in the active cochlear amplification, which depends on cochlear supporting cells and gap junctions^11,12^. Moreover, in our previous study, dominant-negative CX26-R75W transgenic mice that had incomplete development of the cochlear supporting cells and induced no detectable distortion-product otoacoustic emissions, even though the electromotility of isolated OHCs was normal, indicating that normal development of the supporting cells with normal Cx26 was essential for the cellular function of OHCs^10^. From these previous studies, the disruption of cochlear GJPs with aging observed in the present study may result in a reduction in the cochlear OHC amplification rather than in EP, thus causing severe hearing loss even without hair cell loss. We suggest that the degradation of GJPs may not directly cause hair cell loss but may reduce the physiological function of hair cells in the organ of Corti.

**Fig. 7:**
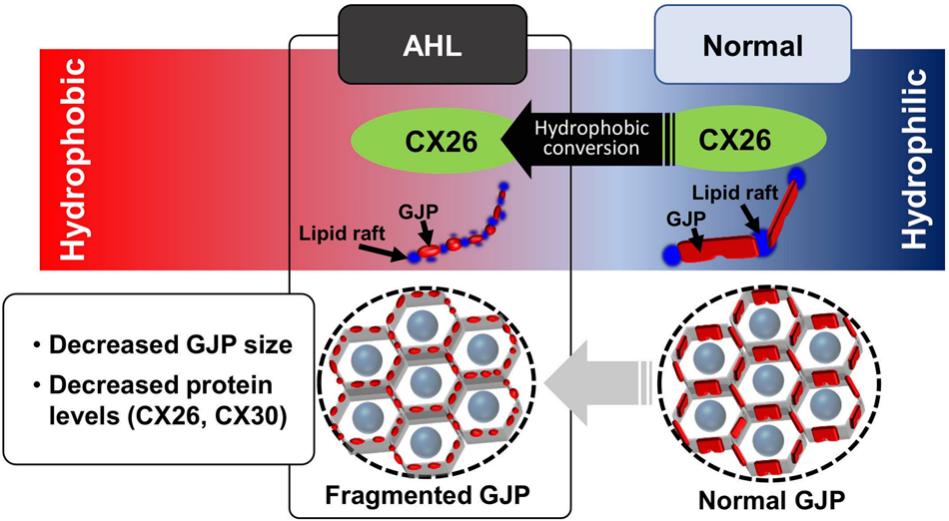
Changes in the biochemical properties of cochlear GJPs in ARHL. In this study, the hydrophilic-to-hydrophobic conversion of Cx26 was observed during the early stage of ARHL. This conversion may lead to a higher affinity of GJPs for membrane lipid rafts, which prevents GJP assembly and reassembly in gap junctions. This may lead to instability and degradation of GJPs, followed by decreased gap junction size and decreased protein levels of Cx26 and Cx30.

Lipid rafts modulate gap-junction-mediated intercellular communication and contain a large quantity of cholesterol and few anionic phospholipids^33,34^. To determine whether the degradation of cochlear GJPs was related to lipid rafts, we investigated cochlear GJPs of ISCs and lipid rafts together by immunofluorescent staining. The orderly arrangement of lipid rafts between GJPs at 4 weeks of age was lost at 32 weeks, when lipid raft staining was diffuse and lipid rafts were distributed in a disorderly fashion around degraded GJPs. This pathological change corresponds to our previous data shown in Cx26-deficient mice^14^, and it is thought that the biochemical property of cochlear GJPs may be converted and become highly associated with lipid rafts in ARHL and *GJB2*-related hearing loss. In the present biochemical study, our results show that Cx26 in the hydrophobic fraction was significantly more enriched in 32-week-old mice than in 4-week-old mice. Schubert et al. investigated the biochemical targeting of connexins to lipid rafts and found that Cx32, Cx36, and Cx46 are efficiently targeted to lipid rafts, whereas Cx26 and Cx50 are specifically excluded from them^35^. On this basis, Cx26 is characterized as being relatively hydrophilic, whereas other connexins are considered hydrophobic. Furthermore, in our LLPS analysis with stable HeLa cell lines overexpressing the wild-type Cx26, Cx26-R75W mutant (dominant negative) or Cx30, the wild-type Cx26 was more enriched in the hydrophilic fraction, although Cx26-R75W and Cx30 were more enriched in the hydrophobic fraction (Kamiya et al., unpublished observation). In addition, Cx26 was targeted to lipid rafts only when Cx26 was coexpressed with Caveolin-1, which is known to play a key role in the aging process^35^. In our previous report, Caveolin-1 was distributed around disrupted GJPs in Cx26 KO mice^14^. This indicated that the expression of aging-related proteins, such as Caveolin-1, might convert to the lipid raft fraction. Increasing the targeting to lipid rafts of connexins occurred to prevent the assembly or reassembly of GJPs in 32-week-old mice. In the present study, our data suggest that, in the C57BL/6J model of ARHL, the biochemical property of Cx26 was converted from primarily hydrophilic in young (4 weeks) cochleae to primarily hydrophobic in cochleae of older (32 weeks) mice. Therefore, we hypothesize that the gradual conversion of Cx26 from hydrophilic to hydrophobic with age is associated with the observed decreases in gap junction size and protein levels of cochlear GJPs (Fig. 7). During the same period, cochlear GJPs were gradually scattered while being invaded by lipid rafts (Fig. 5g–l). Together with the observation of an abnormal lipid raft distribution around disrupted GJPs, our findings suggest that cochlear GJPs in 32-week-old mice may be degraded because of the increased hydrophobic nature of Cx26 and the resulting change in its affinity for membrane lipids.

In summary, our results support the possibility that disruption of GJPs, reduction in gap junction proteins, and biochemical conversion to a more hydrophobic nature might contribute to the development and progress of ARHL. The conversion of biochemical properties of cochlear gap junctions has not been reported in any hearing loss and aging studies, and this might offer a key molecular pathology and new therapeutic target. To the best of our knowledge, this is the first report demonstrating a pathological correlation between ARHL and *GJB2*-related hearing loss at the early pathological stage before significant severe hair cell loss. Furthermore, treatment targeting Cx26, such as the *GJB2* gene therapy we previously demonstrated^36^, may be effective for ARHL.

## Supplementary information

Supplemental Figures

## Data Availability

The data that support the findings of this study are available from the corresponding author upon reasonable request.
