# Supplementary material for: Degradation and modification of cochlear gap junction proteins in the early development of age-related hearing loss
Source: Exp Mol Med. 2020 Jan 27;52(1):166–75. doi: 10.1038/s12276-020-0377-1 (PMC7000393; doi:10.1038/s12276-020-0377-1)
Supplement: Supplementary file 1 — Supplemental Figures [file 12276_2020_377_MOESM1_ESM.pdf]

Supplemental Figure S1.

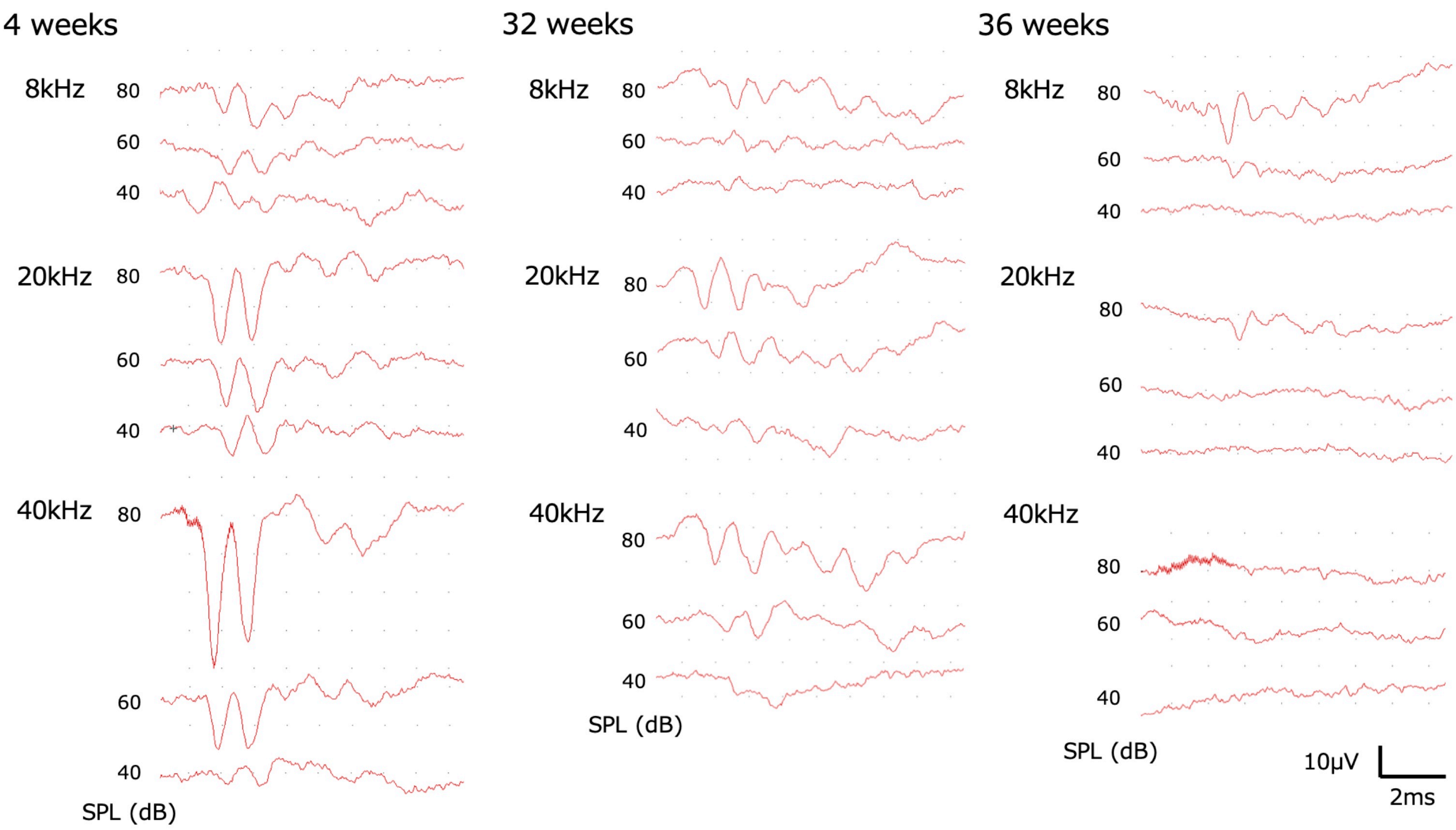

Figure S1. ABR waveforms of 4-, 32- and 36-week-old mice, measured at 40-, 60- and 80dB SPL.

Supplemental Figure S2.

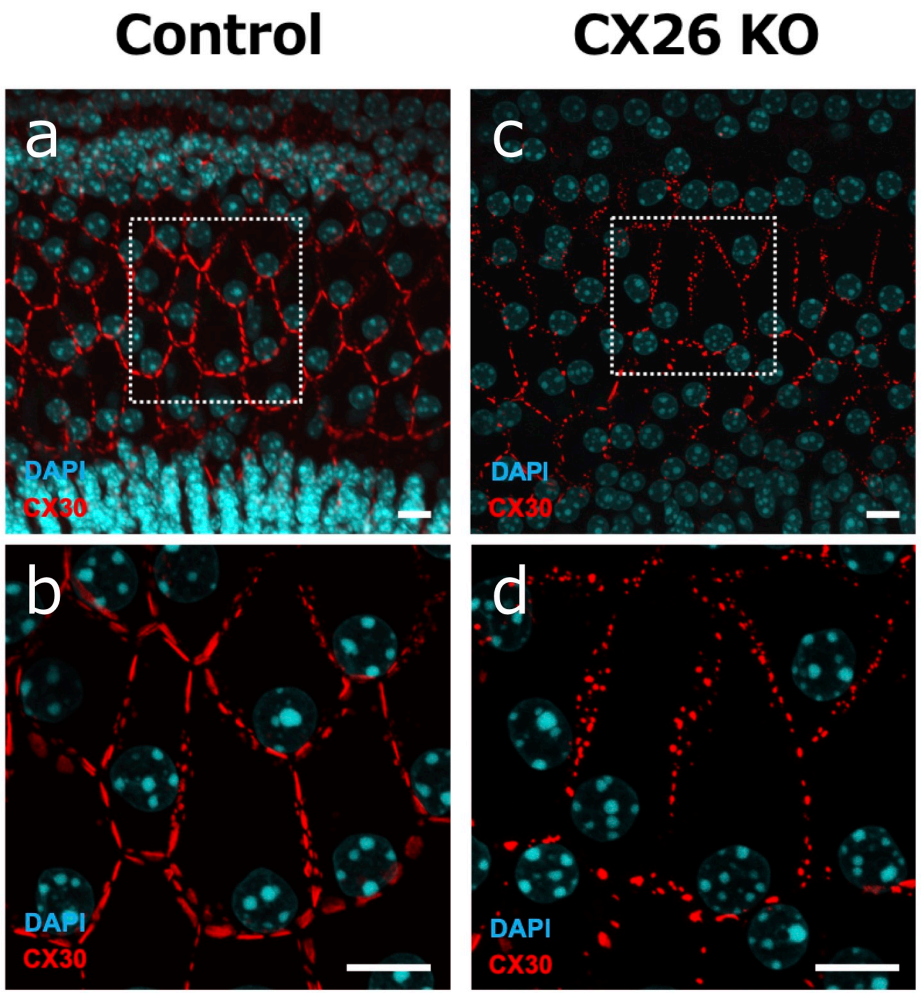

Figure S2. Cochlear inner sulcus cells(ISCs) of CX26 KO mice shows the disruption of GJPs.
Cochlear ISCs from control mice and CX26 KO mice. GJPs in control mice shows normal linear structure. On the other hand, GJPs in CX26 KO mice shows the small spot around the cell-cell junction. Scale bars indicate 10 μm.

Supplemental Figure S3.

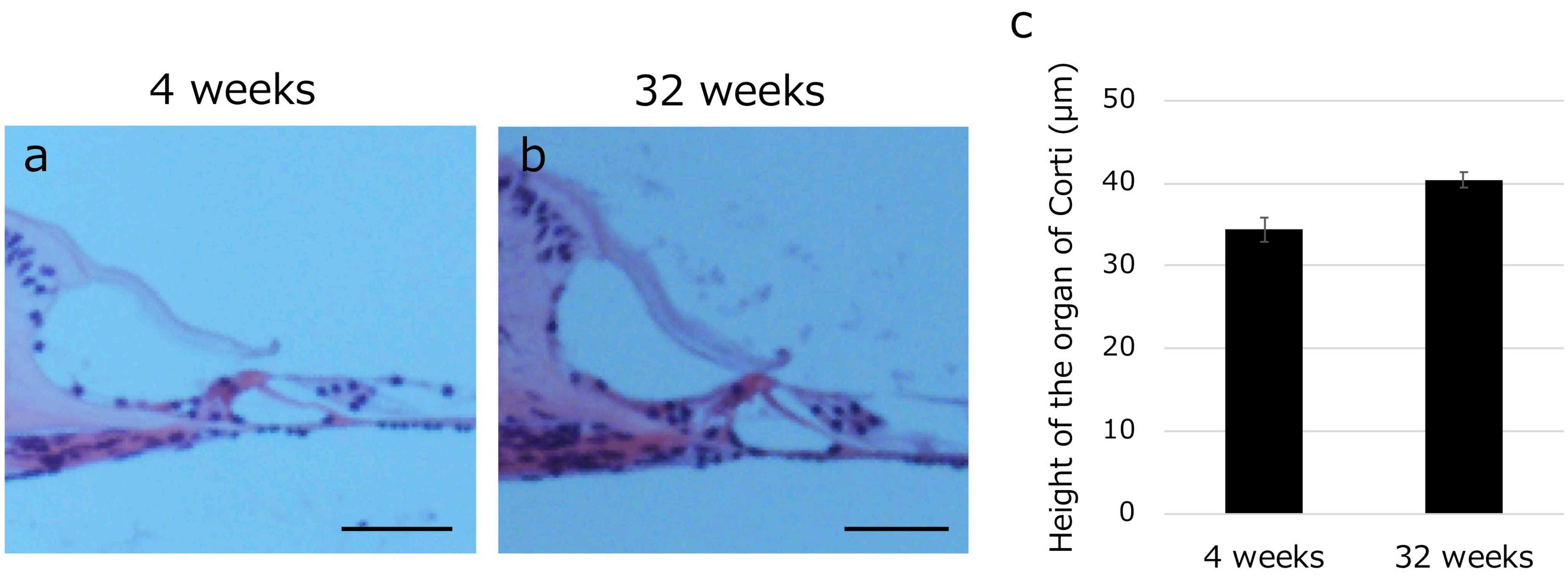

Figure S3. The height of the organ of Corti among 4- and 32-week-old mice.
It seems little morphological change of organ of Corti between 4- and 32-week-old mice. There were no atrophy of organ of Corti between 4- and 32-week-old mice. Scale bars indicate 50 μm.
